# Continuous monitoring system for safe managements of CO_2_ storage and geothermal reservoirs

**DOI:** 10.1038/s41598-021-97881-5

**Published:** 2021-09-27

**Authors:** Takeshi Tsuji, Tatsunori Ikeda, Ryosuke Matsuura, Kota Mukumoto, Fernando Lawrens Hutapea, Tsunehisa Kimura, Koshun Yamaoka, Masanao Shinohara

**Affiliations:** 1 Department of Earth Resources Engineering, Kyushu University, 744 Motooka, Nishi-ku, Fukuoka, 819-0395 Japan; 2 International Institute for Carbon-Neutral Energy Research (I2CNER), Kyushu University, Fukuoka, Japan; 3 Research Center for Negative Emission Technologies, Kyushu University, Fukuoka, Japan; 4 Institut Teknologi Bandung, Bandung, Indonesia; 5 Schlumberger, Clamart, France; 6 Graduate School of Environmental Studies, Nagoya University, Nagoya, Japan; 7 Earthquake Research Institute, The University of Tokyo, Tokyo, Japan

**Keywords:** Geophysics, Seismology, Climate-change mitigation, Energy and society, Sustainability

## Abstract

We have developed a new continuous monitoring system based on small seismic sources and distributed acoustic sensing (DAS). The source system generates continuous waveforms with a wide frequency range. Because the signal timing is accurately controlled, stacking the continuous waveforms enhances the signal-to-noise ratio, allowing the use of a small seismic source to monitor extensive areas (multi-reservoir). Our field experiments demonstrated that the monitoring signal was detected at a distance of ~ 80 km, and temporal variations of the monitoring signal (i.e., seismic velocity) were identified with an error of < 0.01%. Through the monitoring, we identified pore pressure variations due to geothermal operations and rains. When we used seafloor cable for DAS measurements, we identified the monitoring signals at > 10 km far from the source in high-spatial resolution. This study demonstrates that multi-reservoir in an extensive area can be continuously monitored at a relatively low cost by combining our seismic source and DAS.

## Introduction

Carbon capture and storage (CCS) enables us to reduce a large amount of CO_2_ in the near future, and it costs less than many CO_2_ reduction technologies^1–3^. Especially, to achieve negative emissions (i.e., CO_2_ reduction from the atmosphere), the sequestration of the captured CO_2_ into the earth’s geological formation is a key approach^4^. However, reducing a large amount of CO_2_ by CCS to achieve the IEA 1.5 °C scenario (i.e., ~ 15% of the cumulative reduction in CO2 emissions by CCS)^5^ requires thousands of large-scale CO_2_ storage sites (~ 1 million tons/year) in the world. To achieve such a large number of CO_2_ storage sites, we should manage multi CO_2_ storage reservoirs in extensive areas using an innovative monitoring system for the stored CO_2_. Monitoring injected CO_2_ in its reservoir is crucial for predicting the risk of CO_2_ leakage, increasing efficiency, reducing the cost of CO_2_ storage, and reducing the risk of induced seismicity^6,7^. Also, the information derived from monitoring is vital to obtain public acceptance for the projects.

Geothermal power is another main approach to reduce CO_2_ emission using the earth system. In geothermal operations, the elevated pore fluid pressure due to fluid injection often increases seismicity^8^, and reductions in reservoir pressure due to production are monitored to help maintain geothermal operations. Since production and reduction wells in geothermal fields are also widely distributed in the geothermal field, a monitoring system for the multi geothermal reservoirs is crucial for sustainable geothermal power generation^9^ Monitoring, in sum, provides key information for effective and safe reservoir management for CO_2_ reduction. In addition to the CO_2_ storage and geothermal power, the earth monitoring over a spatial range from small reservoirs to the crustal domain is a central technology for energy exploration (e.g., petroleum exploration)^10^, environmental projects (e.g., aquifer utilization)^11^, and disaster prevention (e.g., earthquake fault and volcano monitoring)^12,13^.

In monitoring subsurface reservoirs, we often use elastic properties constrained mainly by seismic velocity^6,14^. Active-source time-lapse (4D) seismic surveys are successfully used for monitoring reservoirs^10^. The temporal and spatial variations of pore pressure or fluid saturation are detected mainly based on variations in seismic velocity. For example, because a *P*-wave velocity dramatically decreases as CO_2_ replaces brine in the pore spaces of reservoir rocks^15^, changes with time in the reflection characteristics of seismic data evaluate the distribution of injected CO_2_^16^. Because of its cost, however, conventional time-lapse seismic monitoring is typically done at long time intervals, making it difficult to identify unexpected and rapid changes in reservoirs (e.g., CO_2_ leakage). For that reason, a continuous monitoring approach has been developed to detect variations of seismic velocity in a timely way. Analyzing the ambient noise in continuous seismometer records shows spatio-temporal velocity variations for monitoring purposes^12,17–19^; in this approach, ambient noise is used to derive virtual active-source seismic data^20^. This method has been used to document crustal-scale seismic velocity variations near earthquake faults and volcanoes^12,21–23^. However, when applying to the shallower reservoirs, the temporal variation of ambient noise characteristics (e.g., variation of its frequency components) decreased the monitoring accuracy^24,25^. These ambient noise characteristics are particularly variable for higher frequencies, strongly influenced by human activities and weather events.

**Figure 1:**
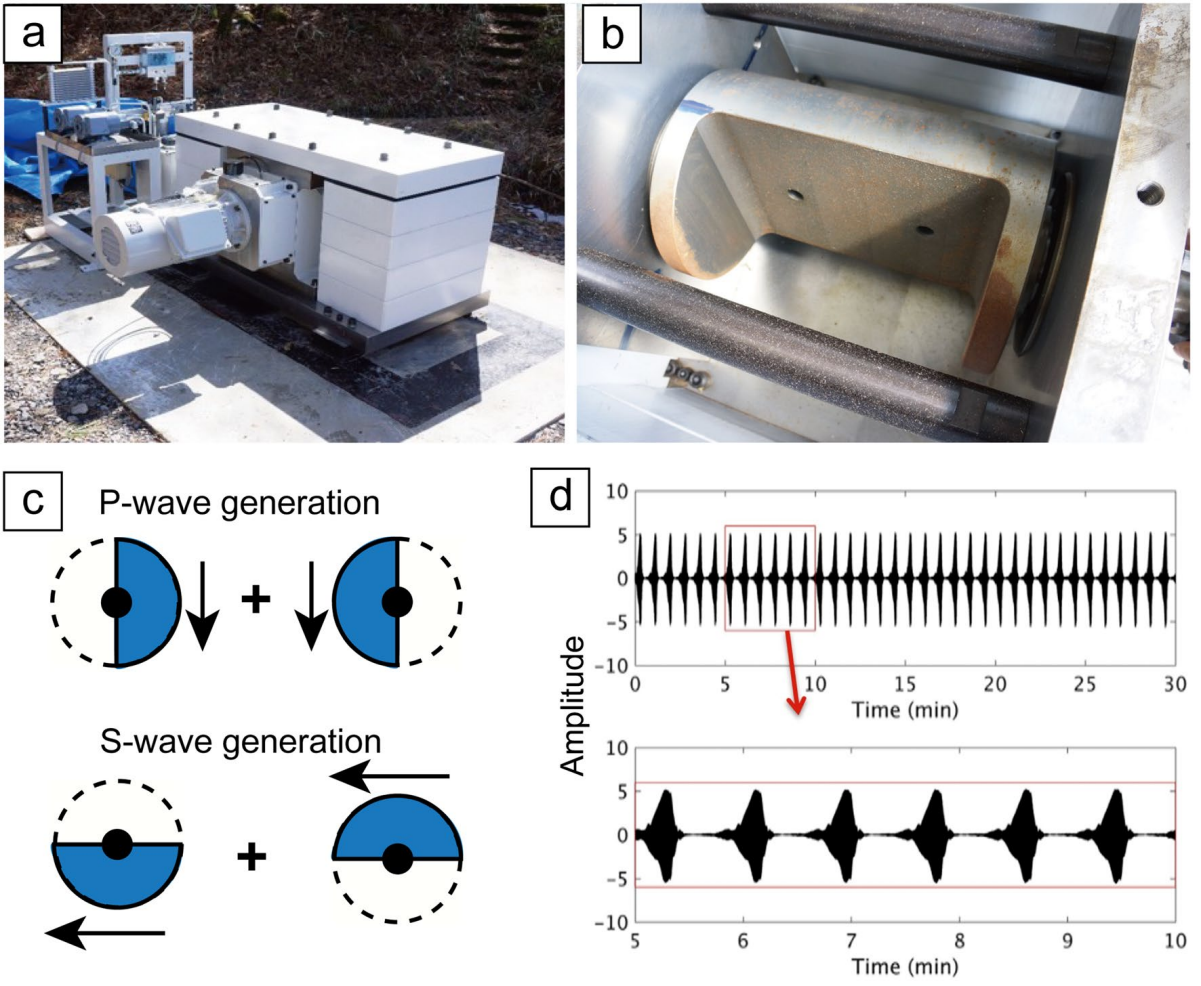
Continuous controlled seismic source system. (**a**) Picture of the continuous source system. (**b**) The eccentric rotating weight of the same source type with larger force. (**c**) Conceptual diagram of vertical and horizontal motions using our monitoring system based on the rotation direction of the eccentric mass. Blue indicates the mass position. (**d**) Chirps generated every 50 s by the source system. The rotation direction is switched every 2 h.

To improve the reliability of monitoring of shallower reservoirs without using ambient noise, we have developed a system that relies on a continuous and controlled seismic source (Fig. 1). In our monitoring source system, repeated signals with a wide frequency range (i.e., chirp) are continuously generated by a rotating eccentric mass^26^. By stacking the continuous waveforms produced by our source system, we can improve the signal-to-noise ratio (SNR) of the seismic signal. Thus, less energetic signals generated from a smaller source can explore deeper geological units. Furthermore, we recorded the source signals with a distributed acoustic sensing (DAS) system based on fiber-optic cables. Because the DAS system enables us to acquire monitoring signals in a long and dense receiver array, our monitoring system shows promise for high-resolution and low-cost monitoring of reservoirs. Here, we report monitoring results for the (1) onshore geothermal field in Kyushu Island in southwest Japan and the (2) offshore geological formation in northeast Japan, using our continuous monitoring source and the DAS system. We discuss the possibility of operating a continuous monitoring system for widely distributed onshore/offshore reservoirs.

## Continuous monitoring system

### Continuous and controlled seismic source

Our permanent seismic source continuously generates repeatable signals (i.e., chirp) with a wide frequency range (10–35 Hz) by a rotating eccentric mass that is precisely synchronized to Global Positioning System (GPS) time (Fig. 1). Stacking the chirps from this source system improves the signal-to-noise ratio of the seismic signal (i.e., transfer function). We calculated the transfer functions (Green’s functions) by deconvolution of the signal recorded at each seismic station with the source function (i.e., chirp) excited by the monitoring source system (Fig. 1d)^27^. If we use different source functions in the monitoring sources, we can operate many monitoring sources in the same field at the same time. To calculate temporal variation of seismic velocity, we compared the transfer function with 1 day or 20 days of stacking (i.e., the current transfer function) to a reference function and calculated the travel time change throughout the period (see “Methods”).

The rotation direction of the eccentric mass in the source system was switched every 2 h. A linear vibration in any direction can be synthesized from a combination of clockwise and counterclockwise rotations. We used the vertical and horizontal components of continuous seismic records to synthesize transfer functions for vertical and horizontal forces (Fig. 1c)^28,29^. A similar continuous-source monitoring system developed for seismology and volcanology, the Accurately Controlled Routinely Operated Signal System (ACROSS)^29^, has successfully monitored changes in seismic properties associated with earthquakes^27,30^, and volcanic activity^31^. However, the ACROSS targets deep features, and its source waveform is dominantly at lower frequencies. To accurately monitor shallower targets, we have downsized the permanent monitoring source system to generate higher frequency waveforms and estimate subsurface behavior at higher resolution. The system we designed in this study uses a rotating mass and generates chirp signals that reach 35 Hz (Fig. 1b). The weight of the mass including the shaft part is ~ 17 kg. The eccentric moment of this mass rotation is 0.507 kg m, and the force during 20 Hz rotation is 8,000 N. Because the vibroseis that is commonly used in time-lapse seismic surveys uses the moving mass with several tons, our seismic source system is much smaller than the conventional source. The surface orbital vibrator (SOV) has been further developed as a similar source system^32–34^. However, our system is larger than the SOV and is designed to monitor multi reservoirs in wider areas (~ 10 km). Also our seismic source system controls the timing of the source signal using GPS, thus we did not need to deploy the seismometer at the source system.

**Figure 2:**
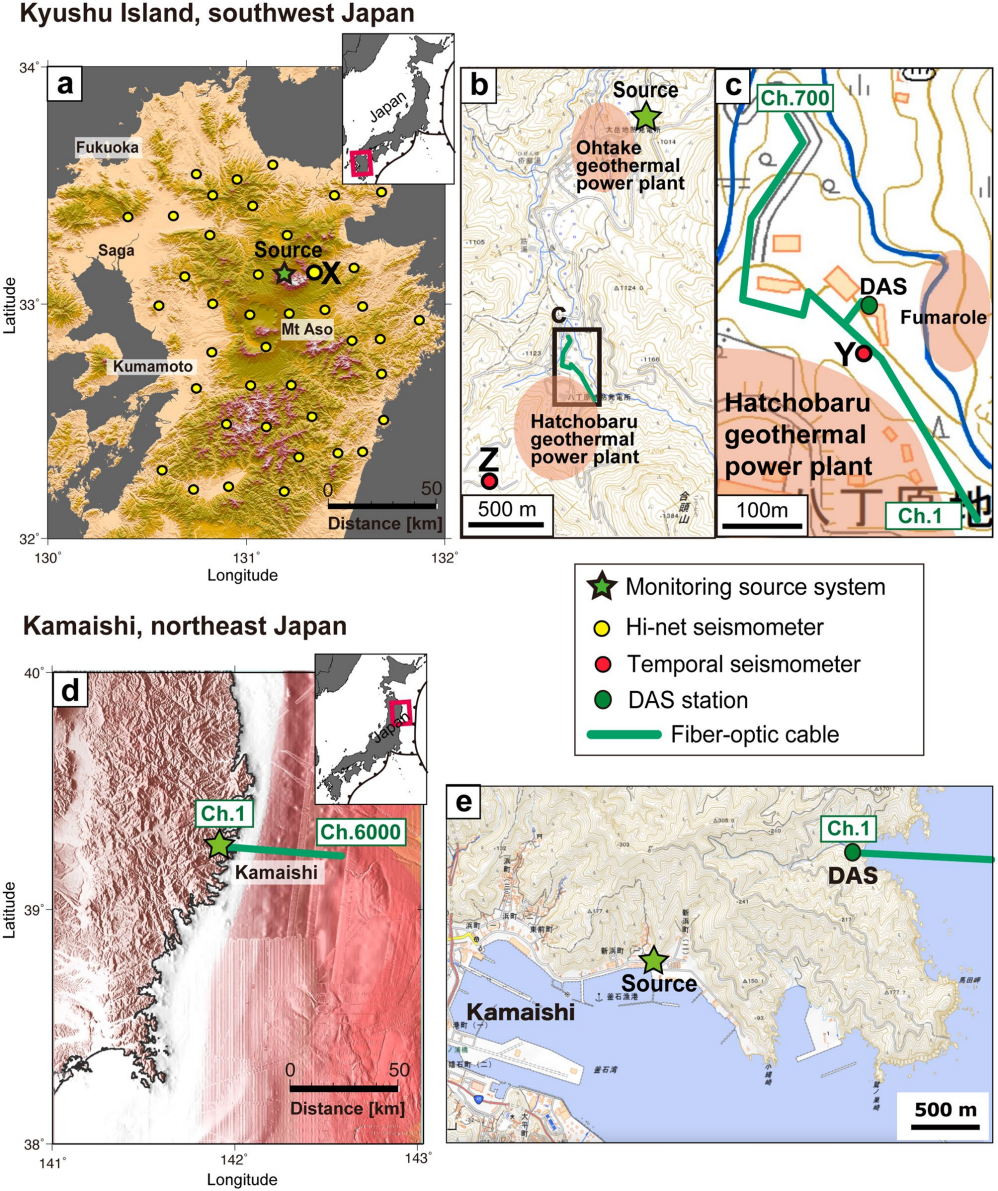
Locations of the continuous monitoring source system, seismometers and fiber-optic cable for DAS. (**a**) Locations of the monitoring source system (green star) and Hi-net seismometers (yellow dots) in the Kuju geothermal field of central Kyushu, southwest Japan. (**b**) Locations of the source system, the temporal seismometers Z (red dot) and fiber-optic cable for DAS (green line). This map includes the locations of 2 geothermal power plants (red shaded areas). (**c**) Positions of the fiber-optic cable (green line) and three-component seismometers Y (red dot). (**d**) Locations of the monitoring source system (green star) and the fiber-optic cable used in this study for DAS (green line) in Kamaishi, northeast Japan. (**e**) Relative locations of source system and fiber-optic cable for DAS. The distance between the source and DAS station is ~ 1.6 km. The base map in panel (**a**) is a 10-m-mesh digital elevation model published by the Geospatial Information Authority of Japan^44^. Panel (**a**) is modified from Nimiya et al.^12^. The base map in panel (**d**) is from JCG and JAMSTEC^45^. We drew the panels (**a**) and (**d**) with Generic Mapping Tools^46^. The base maps in panels (**b**,**c**,**e**) are modified from the Geospatial Information Authority of Japan (http://maps.gsi.go.jp/).

In this study, we have deployed our continuous source system in the Kuju geothermal field in northeastern Kyushu Island, Japan, since 2018 (Fig. 2a–c, Fig. S1), operating with a frequency range of 10.11–20.11 Hz or 12.11–22.11 Hz. To further evaluate the monitoring signal propagation for an offshore fiber-optic cable (i.e., DAS measurement), we deployed the monitoring source system at the ocean coast in Kamaishi city, northeast Japan, in 2020 (Fig. 2d,e, Fig. S1), operating with a frequency range of 12.11–22.11 Hz. We computed the daily transfer functions of the monitoring system by stacking data from 1 day or 20 successive days.

### Long and dense receiver array using DAS

Because deployments of many seismometers improve the spatial resolution of monitoring results, we used a DAS to serve as an array of hundreds/thousands of densely spaced seismometers. Borehole seismic networks based on DAS technology using various fiber-optic cables have been widely used for subsurface imaging^35^. The DAS system can continuously record dynamic strain along the direction of the cable in a succession of ~ 1 m segments for distances of tens of kilometers. This study used the fiber-optic cables deployed at (1) onshore geothermal field and (2) offshore area.

#### Deployment at onshore geothermal field

To evaluate the applicability of DAS for our monitoring source system, we deployed ~ 1.2 km of fiber-optic cable at geothermal power plants at ~ 1.75 km from the monitoring source system (Fig. 2a–c). Because the cable was folded back in some parts, the total survey line is ~ 700 m. The cable was in trenches at a depth of ~ 15 cm to record the horizontal motion parallel to the cable (Figure S2). We recorded the DAS data from 19 September to 5 November 2019. The spatial sample interval or channel spacing was set to 1 m, while the gauge length was 4 m which acts as moving average filter. This experiment used two types of fiber-optic cables, which yielded identical results. The sampling rate was 1 ms in this onshore monitoring experiment.

#### Deployment at offshore area (seafloor cable)

To evaluate whether DAS can be applied to the seafloor cable for offshore monitoring purposes, we used fiber-optic cable deployed on the seafloor off the Kamaishi, northeast Japan (Fig. 2d,e). Many CO_2_ storage projects are suitable for offshore environments, especially in the countries close to the ocean (e.g., Norway, Japan and Indonesia). We recorded the DAS data from 10 October to 23 November 2020. The spatial sample interval was set to 10 m, while the gauge length was 20 m in this test survey. We recorded the seismic signal for the 60 km cable by considering the attenuation of signal propagation of the DAS system. Therefore, we successfully recorded the monitoring signal at 6,000 channels. The sampling rate was 2 ms in this offshore experiment.

## Results and interpretation

### Signal enhancements by stacking

**Figure 3:**
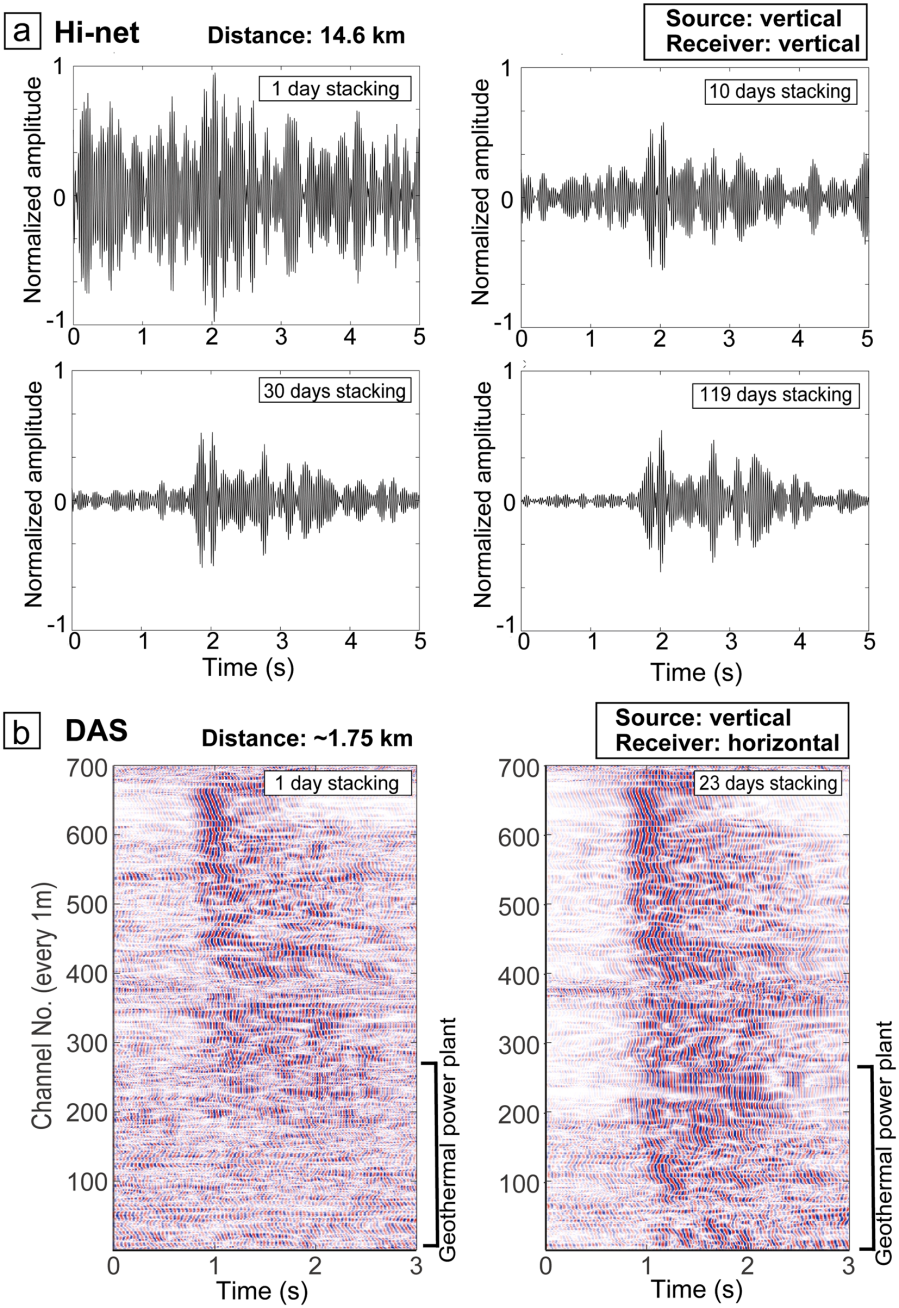
Improvement of signal-to-noise ratio of the monitoring signals by stacking. (**a**) Transfer functions between the source and a Hi-net seismometer for different numbers of stacking days, derived from vertical source motions and the vertical receiver component. The Hi-net seismometer is 14.6 km from the source system (X in Fig. 2a). (**b**) Transfer functions between the source and the 700 channels of the DAS system derived from vertical force data. The high-amplitude waveforms at ~ 1 s represent signals from the continuous monitoring system. The fiber-optic cable is 1.75 km from the source system (green line in Fig. 2b).

By stacking the continuous chirp signals from the source system, random noises cancel each other, and signals are enhanced. The transfer functions between the source and a three-component seismometer located 14.6 km east of the source (X in Fig. 2a) demonstrate that stacking data from longer time periods enhances the signal (Fig. 3a). Because the monitoring signal was distinctly defined in a stack of 10–30 days of data (Fig. 3a), the temporal resolution of the monitoring is ~ 10 days for the seismometer 14.6 km from the source system.

**Figure 4:**
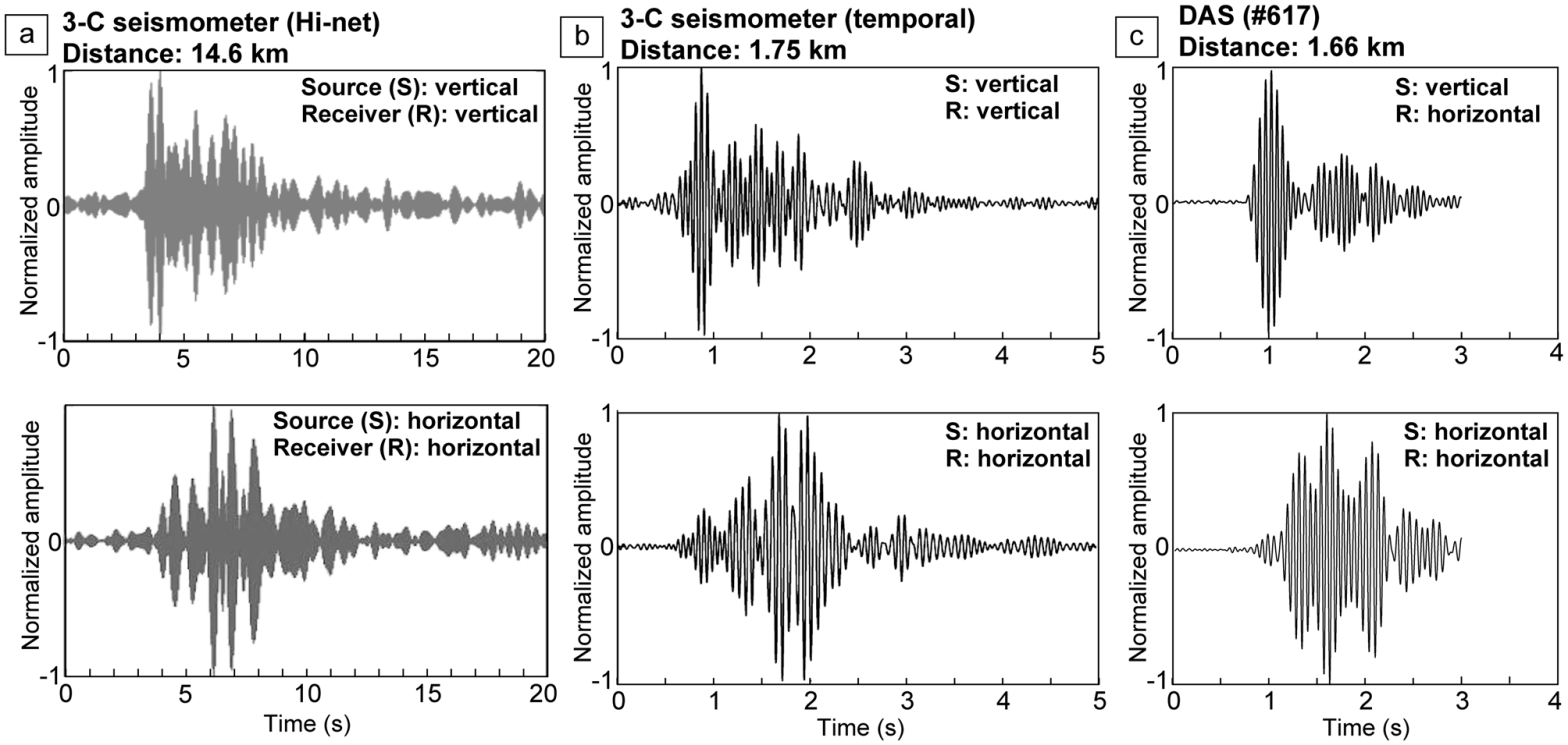
Monitoring signals received by two seismometers and DAS with different distances. (**a**) Transfer functions between the source and a Hi-net seismometer (14.6 km distance). (**b**) Transfer functions between the source and temporal seismometer (1.75 km distance). (**c**) Transfer functions between the source and a DAS record (channel #617). The upper and lower panels show the results from vertical and horizontal source motion, respectively.

Because the seismic source simultaneously generates *P* and *S* waves (i.e., vertical and horizontal motions) by considering rotation directions (Fig. 1c), the records from the three-component seismometers (X in Fig. 2a) yield several wavefields (Fig. 4a). The transfer function derived from the vertical source and receiver records is interpreted as representing *P*-wave propagation, and the transfer function from the horizontal source and receiver could represent the *S* wave. Wavefields derived from other transfer functions include converted waves, such as *PS* converted waves from vertical source motion and horizontal receiver component. The travel times for the first arrivals indicate *P*-wave velocity *Vp* of ~ 4.29 km/s, an *S*-wave velocity *Vs* of ~ 2.36 km/s, and a *Vp*/*Vs* ratio of ~ 1.81. Note that the slightly different ray paths of the *P* and *S* waves add a degree of error to the *Vp*/*Vs* estimate. Using a three-component seismometer close to the source system (1.75 km south from the source system; Y in Fig. 2c), we obtained a similar wavefield (Fig. 4b) and calculated *Vp* as ~ 2.18 km/s, *Vs* as ~ 1.01 km/s, and *Vp*/*Vs* as ~ 2.16. Because the signal propagated through shallower and softer material where *Vp*/*Vs* was higher^36^, the estimated *Vp*/*Vs* (~ 2.16) was higher than that of longer-offset data (~ 1.81).

To evaluate the applicability of the DAS in our monitoring system, we used a fiber-optic cable deployed in the geothermal field (green line in Fig. 2b,c). Because the cable was laid out horizontally in a shallow trench (Figure S2), the DAS system mainly recorded horizontal strain. We demonstrated signal enhancement for the DAS data by the stacking process (Fig. 3b). Although the DAS cable was deployed at a geothermal power plant generating strong noise, we identified the source signal in most channels (1 m segments) of the cable. The signal-to-noise ratio improved with longer stacking periods, but the monitoring signal was clear with a single day of data stacking (Fig. 3b). Therefore, we can monitor at a high temporal resolution (~ 1 day) using a seismometer and a DAS system a few kilometers from the source. The channels with larger noise (channel No. 370) correspond to the locations where the cable did not attach to the ground surface (i.e., bridge). At channel No. 617, the travel time of the vertical source signal was ~ 0.77 s for a P-wave velocity of ~ 2.16 km/s, and the travel time of the horizontal signal was ~ 1.63 s for a S-wave velocity of 1.02 km/s (Fig. 4c). Therefore, the *Vp/Vs* is 2.12. These velocities derived from DAS agree with the 3-component seismometer results shown in Fig. 4b. Although the DAS records could be the horizontal motions, we detected a P-wave from the vertical source motion (Fig. 4c) and estimated the P-wave velocity.

### Signal propagation distance

**Figure 5:**
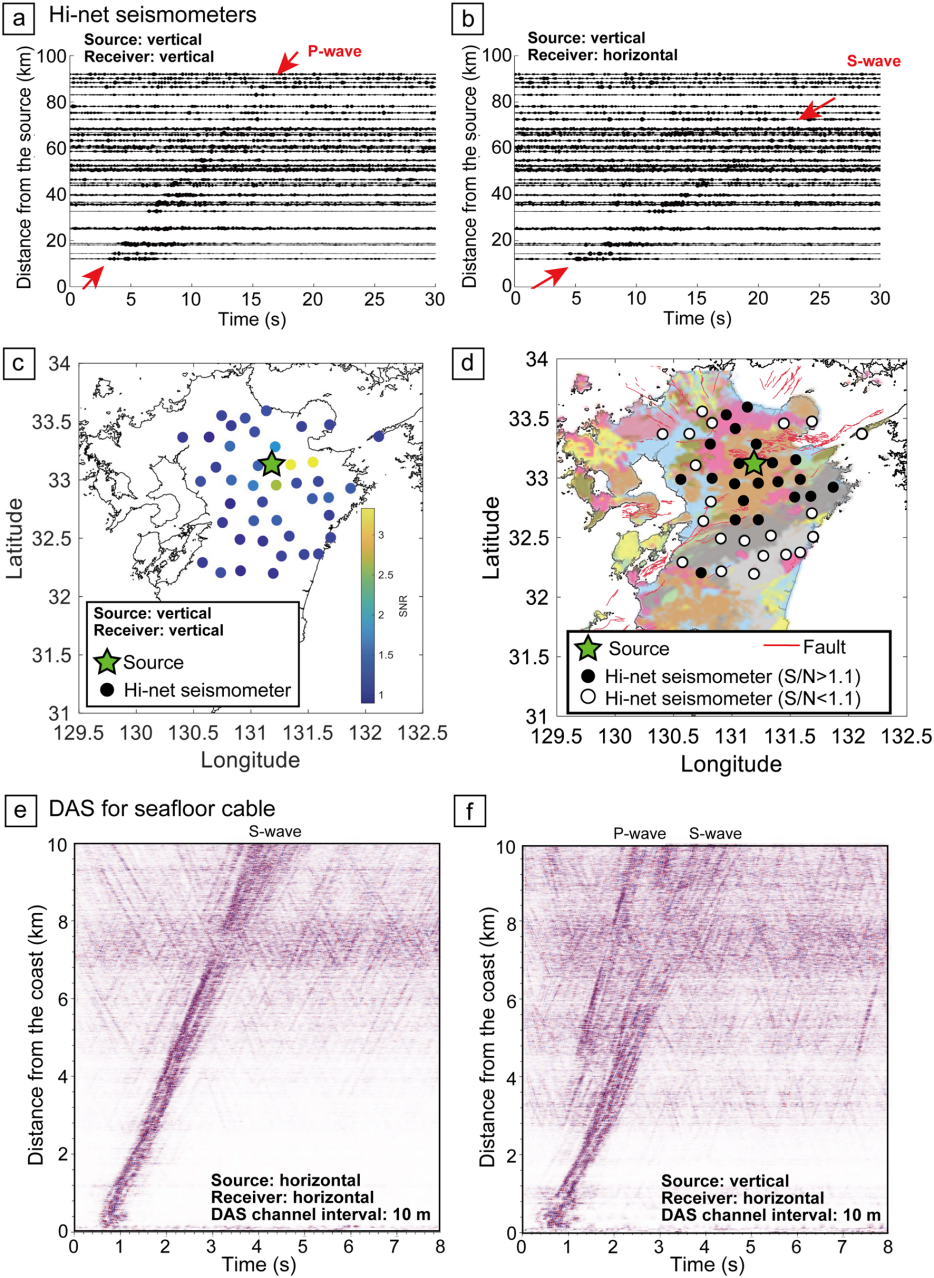
Signal propagation from continuous source system. (**a**) Transfer functions from the vertical source motion and the vertical component of Hi-net seismometers with 2 months of stacking. P-wave was recorded at ~ 80 km distance from the source. (**b**) Transfer functions from the vertical source motion and the horizontal component of Hi-net seismometers. (**c**) Signal-to-noise ratio (SNR) of each seismometer. (**d**) The relationship between lithology and signal-to-noise ratio. The igneous rocks are in pink and brown, and the sedimentary rocks are in other color. The lithological map was from Geological Survey of Japan, AIST, (2015)^44^. We drew the coastline of this map with Generic Mapping Tools^46^. (**e**) Transfer function derived from horizontal motion of the source system and DAS for seafloor cable. (**f**) Transfer function derived from vertical motion of the source system and DAS.

To evaluate the signal propagation for monitoring extensive areas, we analyzed the signal recorded at many Hi-net seismometers in and around Kyushu Island (Figs. 2a, 5). By stacking 4 months of continuous monitoring data with source frequencies of 15.11–20.11 Hz, we successfully observed that the monitoring signal propagated more than 10 km (Fig. 5a,b), although our monitoring source system is small (~ 8,000 N) and generates high-frequency waveforms causing high wave energy attenuation. This long signal propagation could be achieved due to well-controlled continuous signals of our source system. Figure 5a displays the transfer functions (i.e., signal propagation) derived from the vertical source motion and the vertical receiver component, in which the first arrival of the wavefield probably represents the *P* wave. Surprisingly, this first arrival could be identified as far as ~ 80 km away (Fig. 5a), and the *S*-wave arrival ~ 50 km (Fig. 5b). Therefore, we can monitor an extensive area using this source system. We calculated the signal-to-noise ratio of the vertical transfer function for each of the Hi-net seismometer stations (Fig. 5c; see “Methods”). Although the signal-to-noise ratio was high near the source system, there appears to be a lithological effect in that a clearer signal was identified at seismometers deployed in areas of igneous rock east of the source system (Fig. 5d). Where lithological boundaries or fault zones lay between the source and the seismometers, the signal-to-noise ratio appeared lower due to greater wave energy attenuation.

**Figure 6:**
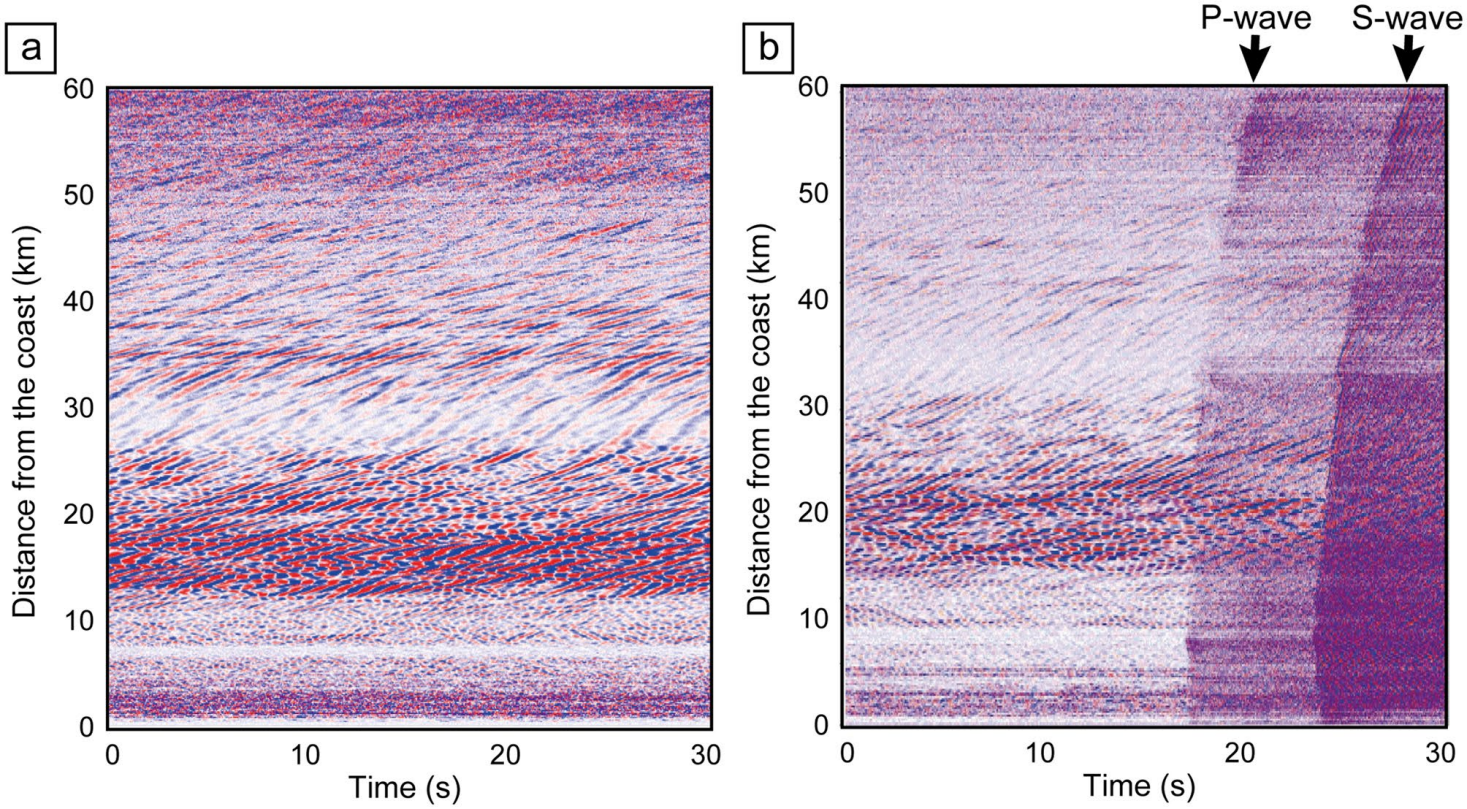
Ambient noise and natural earthquake records by DAS for seafloor fiber-optic cable. Here we displayed 6000 traces with 10 m channel interval. (**a**) Continuous record without the earthquake. (**b**) Continuous DAS record with the earthquake. P-wave and S-wave during the earthquake were clearly recorded at all channels (6000 traces). We clearly observed the noise related to ocean dynamics from 10 to 30 km distance. The location and intensity of the noise is temporarily varied.

We used DAS for the seafloor fiber-optic cable off the Kamaishi to reveal signal propagation in an offshore environment (green line in Fig. 2d). We stacked ~ 1.5-month monitoring data to enhance the signal. Since the signal was unclearer than the onshore experiment, we applied the lateral coherence filter^37^ to enhance the monitoring signal. The unclear signal in offshore environments could be due to the soft sediment at shallow geological formation; the shear motions mainly recorded by DAS are much attenuated within the unconsolidated sediment. After applying the lateral coherence filter and finding optimum deconvolution parameters, we observed the monitoring signal propagation on the offshore DAS records with the distance > 10 km from the source (Fig. 5e,f). Since the strong noises related to ocean-seafloor interaction were observed from 10 to 30 km distance in our DAS record (Fig. 6), we analyzed the monitoring data with the distance < 10 km from the source in this study. When we analyzed the signal from horizontal source motion (Fig. 5e), the signal propagation can be clearly observed. Because the source and receiver are horizontal motion in Fig. 5e, the signal could be S-wave. From the travel time as a function of distance, the S-wave velocity can be ~ 2.5 km/s. The results from vertical source motion (Fig. 5f) enabled us to observe other signal propagations earlier than the S-wave. The signal is related to P-wave, and the velocity was calculated as ~ 4.2 km/s. If the seismic wave is assumed as the P-wave, the Vp/Vs is ~ 1.69 and is lower than the typical Vp/Vs in seafloor sediment^36^. Therefore, there is a possibility that the converted waves are included in the wavefield. If we measure P-wave (vertical motion) using the seafloor cable, the spiral or helical cables that are sensitive for motions perpendicular to the cable could be more suitable.

### Monitoring

We calculated temporal changes in seismic velocity from our monitoring data in the onshore geothermal field (Fig. 2a). In this experiment, we focus on two monitoring periods: (1) 21 October–20 December 2018 (10.11–20.11 Hz source frequency) and (2) 1 October–31 December 2019 (12.11–22.11 Hz source frequency). During these periods, the monitoring source system can be operated stably. During the second period, the geothermal power plant changed the amount of its fluid injection into a reservoir, causing a corresponding change of the pore fluid pressure in the reservoir. In addition, we used the DAS system during a portion of the second period (20 September to 5 November 2019).

**Figure 7:**
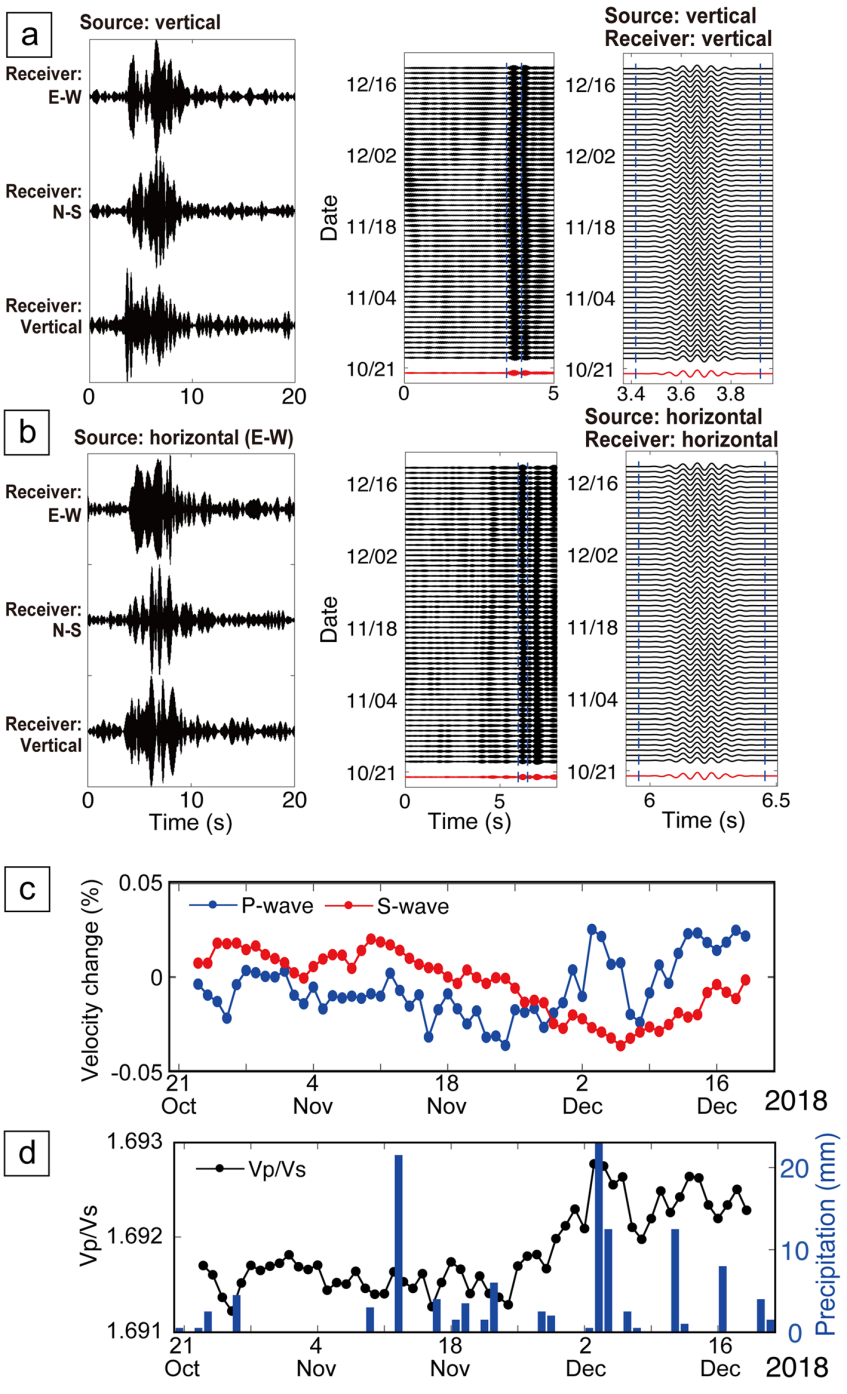
Monitoring results derived from the Hi-net seismometer ~ 14.6 km from the source system. (**a**) Transfer functions derived from vertical source motion (left) and its temporal variation (middle and right). The right panel shows the enlarged waveform close to the first arrival. (**b**) Transfer functions derived from horizontal source motion and its temporal variation. (**c**) Temporal variation of seismic velocity. (**d**) Temporal variation of *V*_*p*_/*V*_*s*_, and precipitation from rain events recorded in the study area.

The results of this exercise are shown in Fig. 7a,b for the transfer function between the source and the 3-component seismometer 14.6 km away. We then calculated the tempral variations of seismic velocity from the transfer function (see “Methods”; Fig. 7c). To stabilize the monitoring result, daily transfer functions were calculated by 20 days of stacking. Because the date of velocity variation shown in Fig. 7c is the central of the 20 days stacking window, the velocity change appears 10 days ahead of the events that influence the velocity change (e.g., rain precipitation). Although the transfer functions were very similar during the monitoring period (21 October–20 December; middle and right panels in Fig. 7a,b), we identified temporal variations of *P*- and *S*-wave velocities with an error of < 0.01% (Fig. 7c). The monitoring results showed that *P-wave* velocity slightly decreased until early December 2018, then increased until mid-December (blue line in Fig. 7c) and that *Vs* decreased until early December and increased after that (red line in Fig. 7c). Furthermore, the *Vp/Vs* was slightly varied from 1.6912 to 1.6927, and it slightly increased after the end of November 2018 (Fig. 7d). These seismic velocity variations are well related to the precipitation (blue bars in Fig. 7d). A positive relationship between *Vp/Vs* and rain may indicate that fluid saturation and/or pore pressure variations influence seismic velocity due to fluctuating groundwater levels. The increase of fluid saturation due to precipitation increases P-wave velocity and decreases S-wave velocity, resulting increase in *Vp/Vs*. Furthermore, the overburden imposed by precipitation (i.e., groundwater level increase) may change the stress state and pore pressure within the deep crust (with low permeability), resulting increase in *Vp/Vs*. We believe that this mechanism (i.e., pore pressure variation) is the most dominant in deeper formation. Similar temporal velocity variation associated with precipitation can be observed by ambient noise analysis^13^. The previous study^13^ revealed a time lag between the precipitation and velocity change; the velocity change occurs several days after the rain events. Therefore, it could be difficult to compare the velocity change and precipitation at same time scale. Also, some other factors such as volcanic activities influence seismic velocity, because the volcano is located between the source and the seismometer.

**Figure 8:**
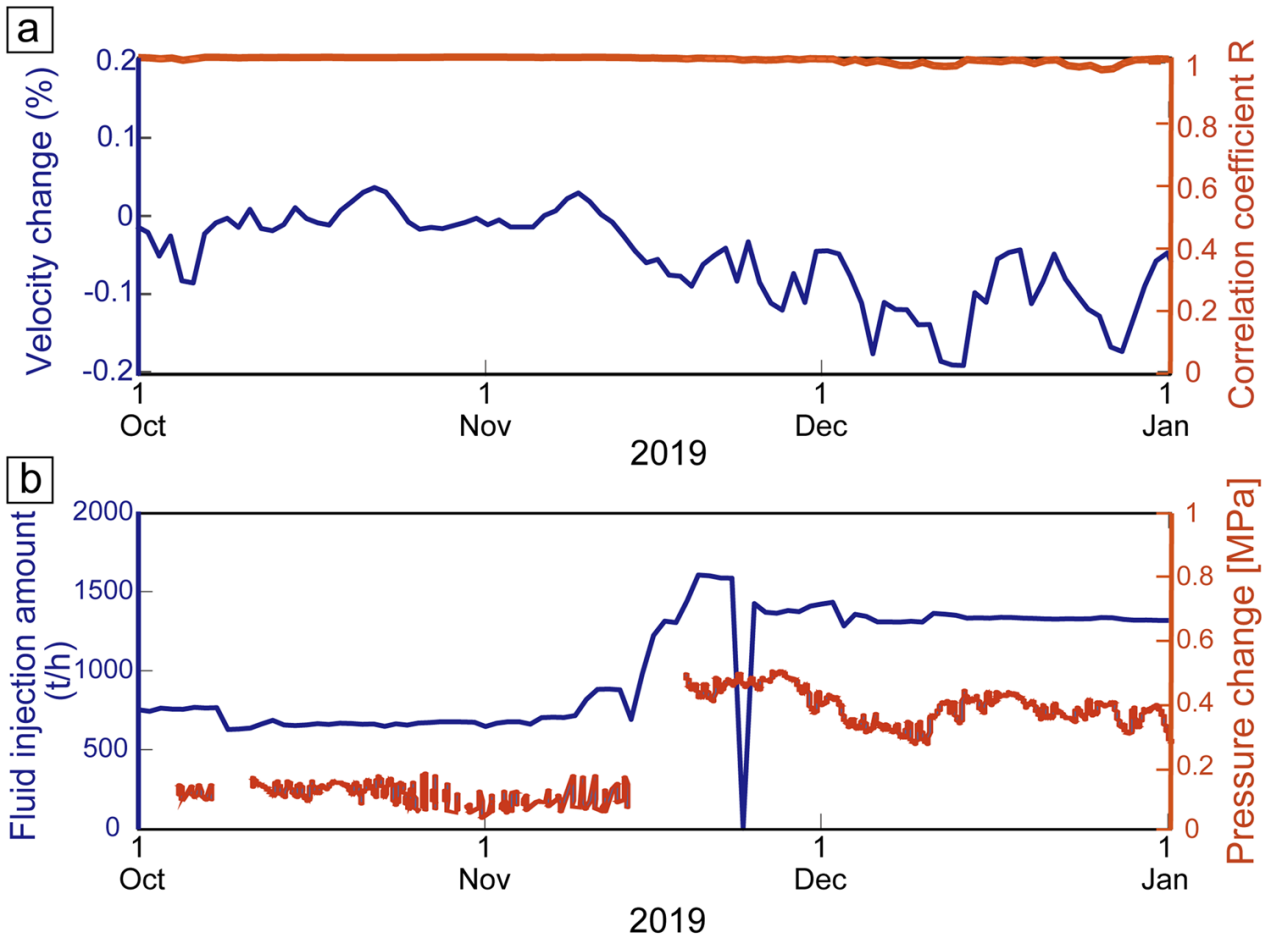
Monitoring results and geothermal operation data during 3 months. (**a**) Temporal velocity variation between the source system and a seismometer ~ 1.75 km (blue), and its correlation coefficient **R** between current and reference trances (orange). The velocity variation is derived from vertical source motion and vertical receiver component (blue). The geothermal fields locate between the source and the seismometer. (**b**) Temporal variations of fluid injection amount (blue) and pressure change in the reservoir (orange).

**Figure 9:**
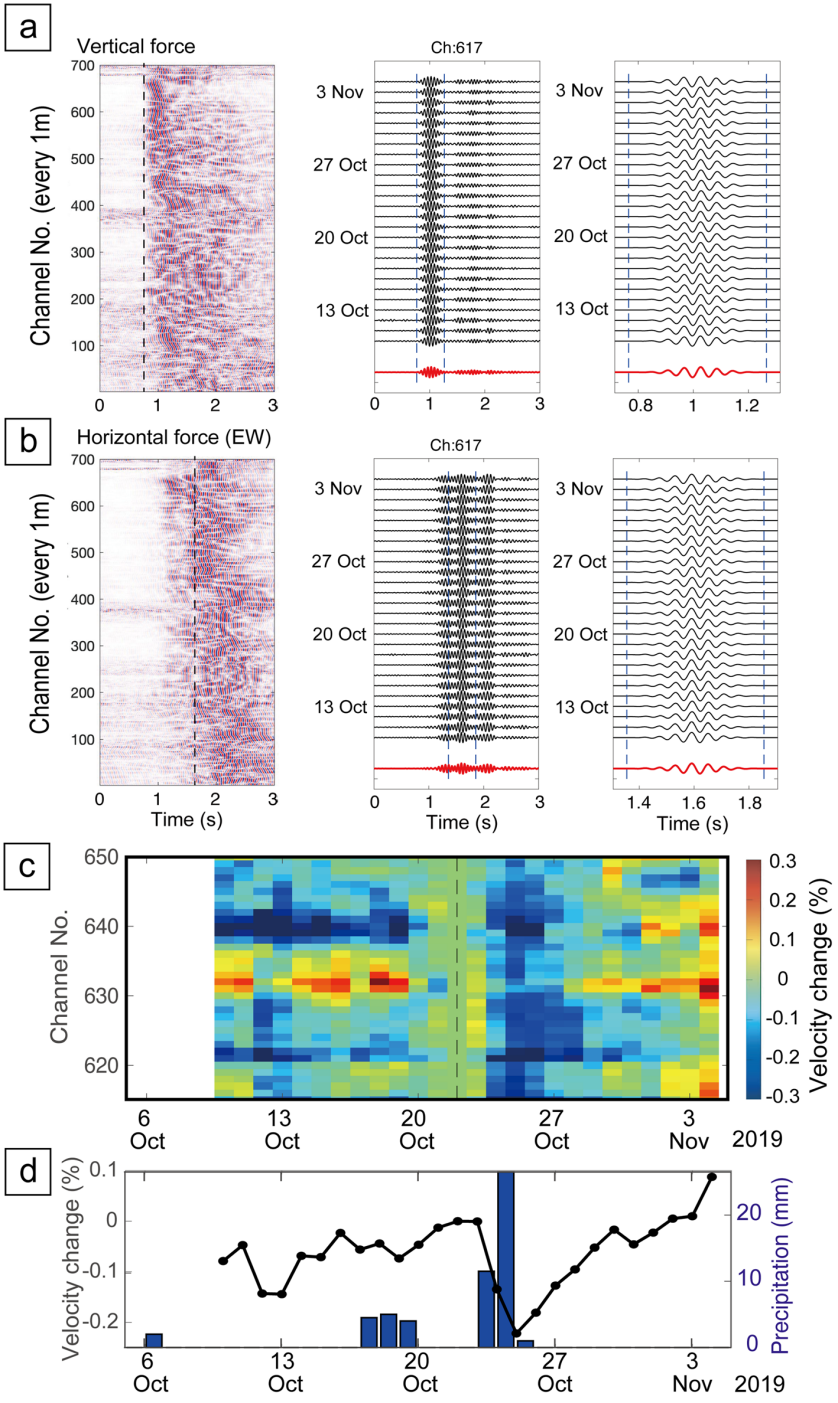
Monitoring results derived from the DAS system ~ 1.75 km from the source system. (**a**) Transfer functions at all receiver channels derived from vertical source motion (left) and temporal variation of channel 617 (middle and right). (**b**) Transfer functions at all receiver channels derived from horizontal source motion and temporal variation of channel 617. (**c**) Temporal variation of P-wave velocity for channels #615–#650 from 10 October to 4 November. The velocity change is defined by using the velocity on 22 October as zero. (**d**) The velocity change averaged from channel #615–#650 results (black), and the precipitation from rain events (blue).

We found velocity variations in short-offset monitoring results via a seismometer (Fig. 8) and DAS (Fig. 9) based on daily stacking of transfer functions. In Fig. 8, we selected the seismometer whose raypath from the source system across the geothermal reservoirs (Z in Fig. 2b). Furthermore, we choose the monitoring period when monitoring source system generated stable signals (from 1 October to 31 December 2019; orange line in Fig. 8a). The longer-period velocity variation (blue line in Fig. 8a) seems to be related to geothermal power plant operations (Fig. 8b). Because the geothermal power plant halted its operation by mid-November 2019 and the injection fluid amount increased after mid-November, the pore pressure in the geothermal reservoir dynamically change; pore pressure close to the reduction wells (or fluid injection well) increased after mid-November (Fig. 8b). Indeed, the P-wave velocity across such reduction areas decreased after starting the plant (mid-November; blue line in Fig. 8a). The P-wave velocity has a negative relationship with pore pressure (or positive relationship with effective stress), thus this relationship can be explained in rock physics models^38,39^. This experiment demonstrates that our monitoring system can reveal temporal variations of pore pressure in the geothermal reservoirs. Because the pore pressure is related to the seismicity^7^, we will be able to use this monitoring system for safe reservoir managements in geothermal power.

The temporal velocity variation acquired by the DAS system had a similar trend in most channels (Fig. 9c), suggesting that DAS provides stable records for the monitoring system. A similar velocity variation can be seen in the seismometer close to the fiber-optic cable. A clear negative correlation between rainfall events and seismic velocity (Fig. 9c,d) can be observed in the DAS record. The seismic velocity variation between the seismic source and the DAS ~ 1.7 km away (± 0.2%; Fig. 9c) was large compared to that in the long-offset monitoring data (± 0.02%; Fig. 7c). Because the ray-path is through shallower formation for shorter-offset monitoring data (shorter distance between source and seismometer; Fig. 9), the velocity variation of the shallower formation is larger than that of deeper crust.

## Discussion and implications

**Figure 10:**
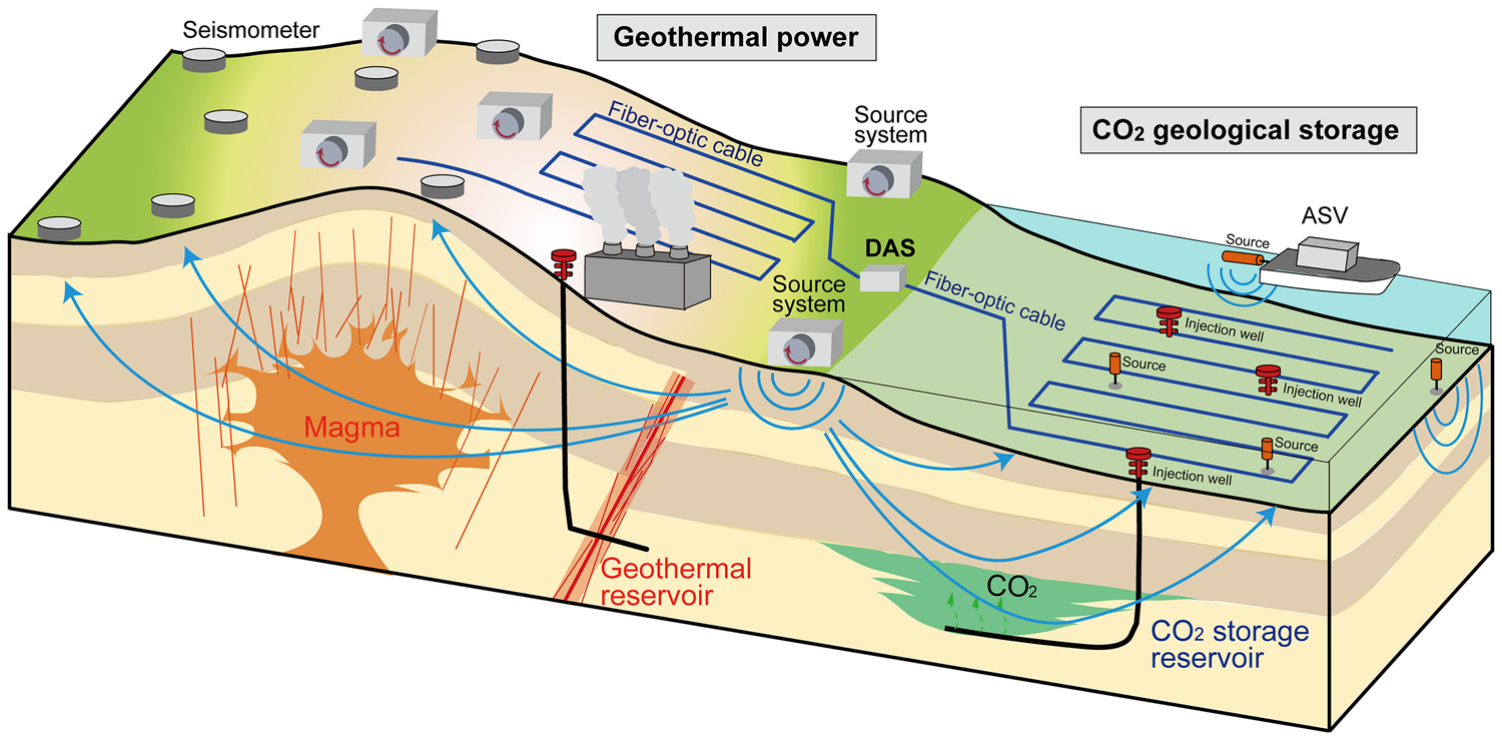
Schematic image of continuous monitoring systems and seismometer networks, including a DAS array. We manage the multi-reservoir using our continuous monitoring system.

We have developed continuous and low-cost monitoring system for multi-reservoir distributed in extensive area (Fig. 10). This study confirmed that the monitoring signal propagates as far as ~ 80 km via onshore seismometers and longer than 10 km via DAS for the seafloor cable (Fig. 5). Because the monitoring data recorded by DAS can be transferred and analyzed in real-time, we can continuously obtain the monitoring results, making it possible to identify unexpected and rapid changes in reservoirs (e.g., CO_2_ leakage). Furthermore, we can operate many monitoring sources in the same field at the same time by using different source functions (Figs. 1d, 10). We measured the temporal variation of seismic velocities with an error of < 0.01% (Fig. 7). The combination of continuous sources and a DAS system (long and dense seismometer array) was sensitive enough to monitor the temporal variation of seismic velocity (Fig. 9). Indeed, we could identify the temporal variation of the geothermal reservoir based on our monitoring system (Fig. 8). The numerical simulation results (i.e., CO_2_ reservoir simulation and dynamic wave propagation simulation) demonstrated that similar monitoring systems can monitor the injected CO_2_ in the reservoirs as seismic velocity variation derived from tomography analysis^40^. Therefore, using our monitoring system, we can monitor extensive areas, such as CO_2_ storage projects or geothermal projects with multiple wells. Because DAS receivers accurately estimate natural earthquakes and CO_2_ injection-induced seismicity, the receiver array via DAS can be also used for such earthquake and geomechanical monitoring^41^. Indeed, our DAS data include several earthquakes, and P-wave and S-wave arrivals of the natural earthquakes can be accurately identified in our DAS record (Fig. 6b). Because our fiber-optic cable is linear-shape in the Kamaishi experiment (green line in Fig. 2d), we cannot accurately estimate the earthquake locations. Nevertheless, if we use a spatially distributed fiber cable as shown in Fig. 10 or use additional seismometers, we can accurately estimate the source locations.

This study showed that smaller source systems (Fig. 1a) could be effective for monitoring multi-reservoir distributed in an extensive area. However, even with several more source systems and dense DAS receivers, the spatial resolution of our system would be inferior to that of time-lapse (4D) seismic reflection surveys^16^. Nevertheless, our monitoring system has value because it does not require frequent expensive time-lapse seismic surveys. The less number of time-lapse seismic surveys could reduce the total monitoring cost. The utilization of the existing seafloor cable for DAS^42,43^ can further reduce the monitoring cost of offshore reservoirs. Because we can continuously monitor reservoirs (e.g., Fig. 8) and immediately detect accidents, this permanent monitoring system may also be valuable for public acceptance in CO_2_ storage and geothermal projects.

In offshore environments, the deployment of our motor-based seismic source system on the seafloor could be difficult, because the soft seafloor sediment cannot hold the source system. Although the monitoring signal propagates for several 10 km from the coast such as the Kamaishi experiment (onshore source and seafloor cable; Fig. 5e,f), it is better to deploy other types of a source in the offshore area in order to improve the spatial resolution of the monitoring results. For example, the sound source sparkers could be deployed close to the seafloor (orange cylinder in Fig. 10). If the source continuously generates the signal with wide frequency, the stacking of the monitoring signal enables us to monitor the deep reservoir, as demonstrated in this study (Fig. 3). To further improve the spatial resolution, we have designed an unmanned vessel (autonomous surface vehicle; ASV) for the monitoring source system (Fig. 10). If the ASV carrying a small seismic source continuously generate the monitoring source signals, we can monitor the offshore reservoirs with higher spatial resolution.

## Methods

### Calculation of temporal variation of seismic velocity

To calculate temporal variation of seismic velocity from our continuous monitoring source system, we compared the transfer function with 1 day or 20 days of stacking (the current transfer function) to a reference function and calculated the travel time change throughout the period using the relationships^30^

1 $$\Delta t=\frac{1}{\sum_{k}C\left({f}_{k}\right)}\sum_{k}C\left({f}_{k}\right)\frac{\theta \left({f}_{k}\right)}{2\pi {f}_{k}}$$

2 $$C\left({f}_{k}\right)=\sqrt{\left|F({f}_{k})\overline{{F }_{ref}({f}_{k})}\right|}$$

3 $$\theta \left({f}_{k}\right)=Arg\left(C\left({f}_{k}\right)\right),$$

where Δ*t* is the travel time change, $C\left({f}_{k}\right)$ is the square root of the amplitude of cross-spectra densities of *k*-th frequency component, $\theta \left({f}_{k}\right)$ is the relative phase delay of cross-spectra densities, *F* is the current transfer function, and *F*_*ref*_ is the reference transfer function. Note that we estimated travel time changes by selecting time windows that included the *P*- or *S-*wave first arrival. Seismic velocity change Δ*v* is estimated from Δ*t* by

4 $$\frac{\Delta v}{v}=-\frac{\Delta t}{t}.$$

This approach can be used when the two transfer functions *F* and *F*_*ref*_ are similar. Therefore, we calculated the correlation coefficient of *F* and *F*_*ref*_ (*R* in Fig. 8a) and used this coefficient to evaluate the reliability of the estimated velocity change.

### Calculation of signal-to-noise ratio

We divided 2 h of monitoring data into 72 segments of 100 s. Because the modulation period was 50 s and two cycles were included in each 100 s time window (Fig. 1d), the estimated amplitude spectra had larger amplitudes for every other data point in the frequency domain, representing signals from the source system. In contrast, the intervening data points corresponded to ambient noise. Therefore, we calculated the signal-to-noise ratio (SNR) for each station by dividing the amplitudes of the signal channel with that of the noise channel. We used the following stacking equations to estimate the levels of signal and noise, then calculate signal-to-noise ratio (SNR):

5 $${\varepsilon }_{sig}=\sqrt{\frac{1}{2{N}_{sig}}{\Sigma }_{{f}_{sig}}{\left|X\left({f}_{sig}\right)\right|}^{2}}$$

6 $${\varepsilon }_{noi}=\sqrt{\frac{1}{2{N}_{noi}}{\Sigma }_{{f}_{noi}}{\left|X\left({f}_{noi}\right)\right|}^{2}}$$

7 $$\mathrm{SNR}={\varepsilon }_{sig}/{\varepsilon }_{noi},$$

where ε is the signal or noise level, *f* is the frequency, *X* is the amplitude in each channel, and *N* is the number of channels. Note the amplitudes of the signal channel |X(f_sig_)| include both signal and noise. When SNR = 1.0, the amplitudes of the signal and noise channels are equal, such that only random noise that exists at all frequencies is dominant. If SNR is significantly higher than 1.0, the signal from the source system can be observed at that station. In Fig. 5d, we showed the seismic station with SNR > 1.1. Overall, the SNR decreases with source-receiver distance (Fig. 5c).

## Supplementary Information

Supplementary Information 1.

## Data Availability

We used Hi-net seismometers opened from the NIED website and the seismometers close to the monitoring source system. The latter seismometers were deployed in the geothermal power plant and cannot be open to the public. The owner of the DAS data is the Ministry of Environment (government of Japan). You may use the DAS data after obtaining approval from the Ministry. The source functions of this continuous source are available from the corresponding author upon reasonable request.
