# Supplementary material for: Continuous monitoring system for safe managements of CO2 storage and geothermal reservoirs
Source: Sci Rep. 2021 Sep 27;11:19120. doi: 10.1038/s41598-021-97881-5 (PMC8476502; doi:10.1038/s41598-021-97881-5)
Supplement: Supplementary file 1 — Supplementary Information 1. [file 41598_2021_97881_MOESM1_ESM.pdf]

Supplementary material

S1. Housing for the continuous monitoring source system

To deploy the continuous monitoring source in the Kuju geothermal field, southwest Japan (**Figure 2a**), we excavated a pit measuring 2.2 m × 2.0 m × 1.0 m and then drilled boreholes for six pillars 3 m long (**Figure S1a**). We poured concrete into the floor of the pit (**Figure S1b**) and fixed a steel frame to this foundation (**Figure S1c**). On the frame, we deployed a coupler for the source system extending to the height of the ground surface (**Figure S1d**) and then poured concrete to the surface level (**Figure S1e**) and deployed the source system on the coupler (**Figure S1f**). The geological formation of this site is not consolidated. It has been continuously used for ~3 years without any problem.

To deploy the source at the coast of Kamaishi, northeast Japan (**Figure 2d**), we made similar-size basement (**Figure S1g-S1i**). Because the consolidated formation is exposed in this area, we could not deploy the pillars to enhance the coupling between the basement and geological formation. We made steel frame for a coupler deployment (**Figure S1h**), and then poured concrete to the surface level for source deployment (**Figure S1i**).

Continuous monitoring system

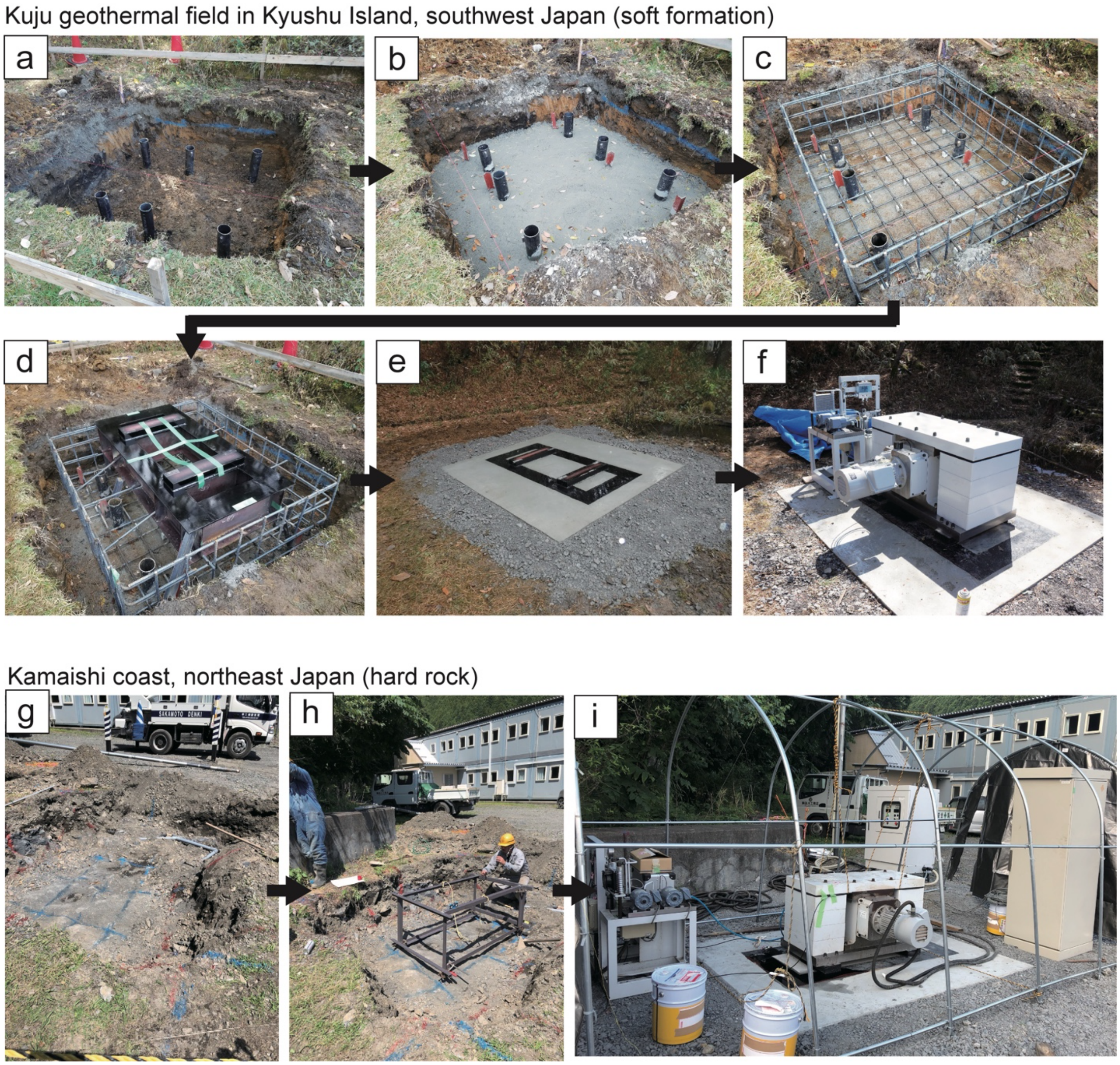

Figure S1. Construction and installation of the continuous monitoring source system in (a)-(f) the Kuju geothermal field, southwest Japan, and in (g)-(i) the Kamaishi, northeast Japan.

S2. Fiber-optic cable deployment

We deployed fiber-optic cable in the geothermal field in a trench ~15 cm deep (**Figure S2**) such that almost all of the cable was buried beneath the ground surface. We deployed the cable without tension (**Figure S2b**). We used two types of fiber-optic cable, but there was no clear difference in their results (**Figures 3b, 9a, 9b**).

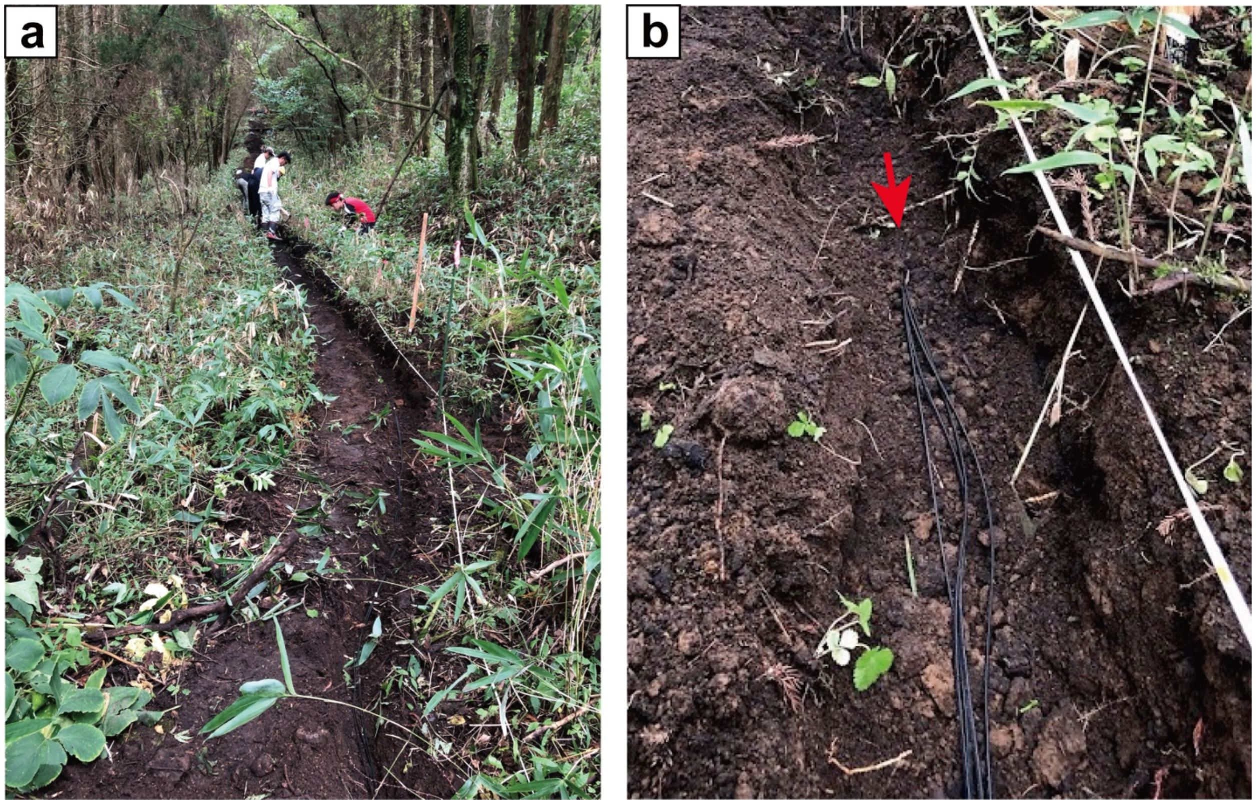

Figure S2. Deployment of fiber-optic cable in the Kuju geothermal field, northeast Kyushu, Japan. (a) The trench. (b) Deploying cable.
